# Cure rate of infections is not an argument for spacer in two-stage revision arthroplasty of the hip

**DOI:** 10.1007/s00402-022-04463-9

**Published:** 2022-05-09

**Authors:** Dominik Adl Amini, Chia H. Wu, Carsten Perka, Henrik C. Bäcker

**Affiliations:** 1 Department of Orthopedic Surgery and Traumatology, Charité, University Hospital Berlin, Charitéplatz 1, 10117 Berlin, Germany; 2 Department of Orthopedics & Sports Medicine, Baylor College of Medicine Medical Center, Houston, TX USA

**Keywords:** Infection, Total hip arthroplasty, Girdlestone, Complications, Spacer, Cure rate

## Abstract

**Introduction:**

A devastating complication after total hip arthroplasty (THA) is chronic periprosthetic joint infection (PJI). Most frequently spacers (Sp) with or without antibiotics are implanted in a two-stage procedure even though not always indicated due to unknown pathogen, femoral and acetabular defects or muscular insufficiency.

**Materials and methods:**

A retrospective analysis of a prospectively collected database was conducted, analyzing the treatment of 44 consecutive cases with chronic PJI undergoing two-stage revision using a Girdlestone situation (GS) in the interim period between 01/2015 and 12/2018. Diagnostics included intraoperative microbiological cultures, histological analysis, sonication of the initial implant, analysis of hip aspiration, as well as laboratory diagnostics and blood cultures. We analyzed the general and age-group-specific success rate of treatment using GS. Furthermore, we compared our data with the current literature on spacer implantation regarding common complications.

**Results:**

In total, 21 female and 23 male patients at a mean age of 59.3 ± 9.6 years were included. Age groups were divided into young, mid-age, and elderly. In most patients, microbiology revealed Staphylococcus epidermidis in 39.1% of cases, following *Staphylococcus lugdunensis* and *Staphylococcus aureus* in 10.9% after THA explantation. For histology, Krenn and Morawietz type 2 (infectious type) was diagnosed in 40.9%, type 3 (infectious and abrade-induced type) in 25.0%. With GS, the total cure rate was 84.1% compared to 90.1% (range 61–100%) using Sp as described in the literature. Among age-groups, cure rate varied between 77.8 and 100%. Other complications, which only occurred in the mid-age and elderly group, included the necessity of transfusion in 31.1%, and in total, one periprosthetic fracture was identified (2.3%).

**Conclusion:**

GS shows an acceptable cure rate at a minimum of 2 years when compared to the cure rate reported in the literature for Sp without major complications. For patients with increased risks for treatment failure using spacer, GS seems to be an alternative for chronic PJI when looking at the success rate of treatment.

**Level of evidence:**

III, Retrospective trial.

**Supplementary Information:**

The online version contains supplementary material available at 10.1007/s00402-022-04463-9.

## Introduction

One of the most popular orthopedic procedures is the total hip arthroplasty (THA) [1–3]. With an increasing number of primary surgeries, complications such as periprosthetic joint infection (PJI) will be more common which require revision procedures, since PJI is a life-threatening disease with a high mortality (range from 7 to 62%) [4–6].

Up to now, a variety of different salvage techniques have been described. Nowadays, most frequently spacers (Sp) with or without antibiotics are implanted in a two-stage procedure even though not always indicated due to unknown pathogen, femoral and acetabular defects or muscular insufficiency. Alternatives include a Girdlestone arthroplasty (GS), or in rare cases, a single-stage procedure [4, 7]. Both Sp and GS have their own advantages and disadvantages.

For GS, the greater dead space and soft-tissue contracture that consequently might lead to leg length shortage due to a hamper reimplantation of the new prosthesis, are described. This might cause worse functional outcomes and an adverse effect of the patients’ quality of life [8]. However, the incidence is unclear as well as the differentiation among age. For Sp, dislocation, bone fractures and implant failure are the main complications [9, 10]. Furthermore, the successful treatment using Sp differs by multiple factors like the type of spacer (handmade, molded or prefabricated), the general geometry especially the head/ neck ratio, mismatch between head of the spacer and the acetabular diameter, bone quality of the patient, and many more [7]. Moreover, when using Sp, the successful treatment can differ according to the used bone cement. When using polymethyl methacrylate (PMMA), a colonization with pathogens in up to 50% can be observed which may require an extended systemic antibiotic therapy [11]. Although Sp can be loaded with antibiotics, the surface can facilitate bacterial growth causing the development of a biofilm which then may cause bacterial resistance.

This study aims to analyze (1) the successful cure rate when performing GS and (2) the variation among age-groups as well as (3) comparing the most common complications of Sp as described in the literature with the complication of GS as used in our single-center specialized in septic surgery.

## Materials and methods

### Study design and population

A retrospective analysis of a prospectively collected database was conducted between January 2015 and December 2018. In total, 44 patients below the age of 70 years matched our inclusion criteria and were diagnosed with chronic PJI of the hip. All patients were treated by a standardized comprehensive therapeutic and diagnostic algorithm with two-stage revision surgery using GS.

Inclusion criteria consisted of (I) patients below the age of 70, (II) patients with chronic periprosthetic joint infection after total hip arthroplasty following the Musculoskeletal Infection Society (MSIS) classification for periprosthetic joint infection [12–14]. (III) patients undergoing full two-stage exchange surgery at our institution, IV) as well as patients with a minimum follow-up of 24 months after reimplantation. Patients were excluded with (I) native infected joints, (II) acute PJIs with onset of less than 6 months after primary THA, (III) follow-up of less than 24 months, (IV) no intention of reimplantation, and (V) violation of the treatment protocol were excluded from this study.

Internal review board approval was obtained by the institutional ethics committee (EA4/201/19) and the study was performed in accordance with the Declaration of Helsinki.

### Data collection

Patient demographics, including age at surgery, gender, comorbidities, route of infection, the patients’ medical history, surgical records, microbiological and histopathological records (hip synovial aspiration, blood cultures, intraoperative tissue samples, and sonication of the initial implant), as well as laboratory values (C-reactive protein (CRP), blood leucocytes), were obtained by reviewing electronic medical charts. Age groups were classified into young (under the age of 44 years), middle aged (between 45 and 57 years), and elderly patients (between 58 and 70 years). All medical reports, adjunctive reports, and pre- as well as postoperative radiographies were analyzed by an arthroplasty trained orthopedic surgeon.

### Diagnostic algorithm

All patients underwent the same diagnostic and surgical algorithms. For follow-up physical examination, laboratory tests including c-reactive protein (CRP) and white blood cell count (WBC) as well as plain anteroposterior and lateral radiographs were performed. Diagnostic joint aspiration was carried out for suspected chronic PJI. According to the MSIS classification, synovial fluid leucocyte count of 3.000/mm^3^ or more than 80% granulocytes were considered as a chronic PJI [15, 16]. For histology, the Morawietz histopathological classification was applied and positive histopathological results were stated for type 2 (infectious type) and 3 (infectious and abrade-induced type) periprosthetic membrane [17]. All tissue samples and blood cultures were incubated for a minimum of 14 days and antibiogram showing antibiotic resistance by the microbiology laboratory.

### Surgical algorithm

A two-stage revision procedure was performed in all patients using GS as described earlier [16]. During the stage-one surgery, the previous approach is used or if present an approach along a fistula, to remove all implants and foreign materials as well as necrotic tissue. The initial total hip implant was sent for sonication and a minimum of five microbiological and one pathological sample was taken as well as generous debridement, and pulse lavage was carried out. Intravenous antibiotic treatment (see below) was adjusted interdisciplinary prior surgery with a specialist for infectious disease and initiated directly after gaining tissue samples intraoperatively. In the case of a patient presenting signs of sepsis, preoperatively intravenous antibiotic treatment was started after synovial aspiration. Based on a previously published concepts [15, 16], each patient underwent an empirical intravenous antimicrobial therapy postoperatively after the stage-one surgery for approximately 2 weeks. This included Amoxicillin Calvulanate and Vancomycin and was adjusted once the microbiological results revealed the pathogen in consultation with the specialists for infectious disease. For the definite antimicrobial protocol, no standardized protocol was followed. Typically, intravenous antibiotics were applied for 2 weeks followed by oral antibiotics with high oral bioavailability until reimplantation of the THA. Additionally, physiotherapeutic treatment was initiated from the first postoperative day onwards with 15 kg partial weight-bearing according to the patient’s resilience.

Afterward, without further evidence of an ongoing infection and decreased CRP levels, the patients were discharged from our hospital with oral tailored antibiotics according to the susceptibility of the isolated pathogen until the date of re-admission for reimplantation. Regular wound controls were carried out prior reimplantation. Reimplantation was indicated in patients with a (I) good general health condition, and (II) a healed stage-one wound, (III) no drainage, redness or increased swelling, and (IV) presence of laboratory signs of controlled infection (continuously decreasing C-reactive protein) [8, 16]. Hereby, microbiological and pathological samples were taken again as in stage-one surgery and pulse lavage, as well as debridement was carried out a second time to minimize the risk of re-infection. Postoperatively, an intravenous antibiotic treatment was again initiated after reimplantation for 2 weeks according to the previously present pathogen. Afterward, tailored oral antibiotics according to the recommendations of our interdisciplinary specialist for infectious disease was given for 5 weeks after discharge of the patients. Treatment success was evaluated according to the Delphi international multidisciplinary consensus [18, 19].

Furthermore, treatment was considered as successful if all of the following criteria were fulfilled at the 24 month follow-up as previously reported in the literature [16]:

1. Healed wound without a fistula, drainage, or pain.
2. The absent of additional subsequent surgical intervention for infections.
3. No recurrent infection caused by the same pathogen.

### Comparison between spacer and girdlestone

To assess data on success rate and complication rate after spacer implantation, a systematic literature review was performed following the PRISMA guidelines. [20] Therefore, PubMed, MEDBASE, Cochrane, and Google Database were searched for ‘PERIPROSTHETIC INFECTION’ and ‘HIP’ and ‘SPACER’. Data regarding the usage of Sp in two-stage revision procedures were analyzed and finally compared with our data using GS.

## Results

In our cohort, in total, 21 female and 23 male patients at a mean age of 59.3 ± 9.6 years at time of surgery matched our inclusion criteria. In 81.8% (36 patients), infection was assumed to occur perioperative at implantation, whereas in 18.2% (8 patients), it was considered hematogenous. The mean CRP at admission was 48.1 ± 66.0 mg/l and the mean stay in hospital during stage-one surgery was 17.9 ± 10.2 days. For stage-two surgery (reimplantation of the new prothesis), the mean hospital stay was 14.4 ± 7.1 days. In total, the mean prosthesis-free interval was 69.0 ± 34.2 days. Mean duration of antibiotic therapy in days was 63.0 ± 29.3 for stage-one surgery and 68.0 ± 88.4 for stage-two surgery (Table 1).

**Table 1:** Patient characteristics

Variable	*n*
Gender (%)	
Female	21 (47.7)
Male	23 (52.3)
Mean age at stage-one surgery (SD)	59.3 (9.6)
Mean hospital stay in days (SD)	
At stage-one surgery	17.9 (10.2)
At stage-two surgery	14.4 (7.1)
Microbiology at stage-one surgery (%)	
Monomicrobial	29 (65.9)
Polymicrobial	9 (20.5)
Negative	6 (13.6)
Microbiology at stage-two surgery (%)	
Monomicrobial	5 (100)
Mean CRP at admission in mg/l (SD)	48.1 (66.0)
Mean duration of antibiotic therapy in days (SD)	
At stage-one surgery	63 (29.3)
At stage-two surgery	68 (88.4)
Mean prosthesis-free interval in days (SD)	69 (34.2)

### Microbiological and histological findings

The results of the microbiological cultures and sonication can be found in Table 2. In total, 9 patients (20.5%) showed infections with multiple pathogens. No bacterial growth was observed in 6 (13.6%) patients, but due to the elevated cell count, these cases were treated as culture negative infections. In total, 5 pathogens were detected after reimplantation including one patient presenting the same pathogen as at explantation. This patient was considered as a failure of treatment as described above. For histology, Morawietz type 2 (infectious type) was diagnosed in 40.9%, type 3 (infectious and abrade-induced type) in 25.0%, type 1 (abrade-induced type) in 9.1%, and type 4 (non-infectious and non-abrade-induced type) in 6.8%.

**Table 2:** Microbiological cultures and sonication

	Explantation		Reimplantation
	*n* (%)	*n* (%)	*n* (%)
	46 (100%) Microbiology	32 (100%) Sonication	5 (100%) Microbiology
Gram positive	44 (95.7%)	31 (96.9%)	5 (100%)
*Staphylococcus epidermidis*	19 (39.1)	12 (37,5)	2 (40%)
*Staphylococcus lugdunensis*	5 (10.9)	3 (9.4)	1 (20%)
*Staphylococcus aureus*	5 (10.9)	3 (9.4)	–
*Enterococcus faecalis*	3 (6.5)	3 (9,4)	–
*Propionibacterium acnes*	3 (6.5)	2 (6.2)	–
*Staphylococcus hominis*	2 (4.4)	–	–
*Staphylococcus capitis*	2 (4.4)	1 (3.1)	–
*Streptococcus dysgalactiae*	1 (2.1)	1 (3.1)	–
*Streptococcus agalactiae*	1 (2.1)	1 (3.1)	–
*Staphylococcus haemolyticus*	1 (2.1)	2 (6.2)	–
*Bacillus cereus*	1 (2.1)	–	1 (20)
*Bacillus simplex*	1 (2.1)	–	–
*Streptococcus mitis/oralis*	1 (2.1)	1 (3.1)	1 (20)
*Staphylococcus warneri*	1 (2.1)	2 (6.2)	–
*Propionibacterium avidum*	–	1 (3.1)	–
*Staphylococcus auricularis*	–	1 (3.1)	–
*Gram negative*	2 (4.3)	1 (3.1)	
*Pseudomonas aeruginosa*	1 (2.2)	1 (3.1)	–
*Paracoccus yeei*	1 (2.2)	–	–

### Successful treatment and complication

After a minimum of 24 months, successful treatment following two-stage revision procedure for the treatment of periprosthetic hip infection was observed in 84.1% (37 out of 44 cases). For the remaining 15.9%, one patient required a second girdlestone arthroplasty after reimplantation of the new prosthesis. This patient had no bacterial growth at stage-one surgery, but due to the elevated cell count, this case was treated as culture negative infection. Microbiological and pathological samples were taken again in stage-two surgery which showed growth of *Staphylococcus aureus* at reimplantation. Another case was considered as a failure due to a recurrent infection with the same pathogen (*Staphylococcus lugdunensis*) and 5 cases needed long-term (> 6 month) antibiotic suppressant treatment. No further revision surgery was required in these cases.

When looking for the individual cure rate among age, we found a substantial higher rate between 32 and 44 years of age (100%) in comparison to the mid-aged and elderly patients (77.8% and 83.9%). Treatment failure in mid-aged group contained 1 case due to a recurrent infection with the same pathogen (*Staphylococcus lugdunensis*) and 1 case with the necessity of long-term (> 6 month) antibiotic suppressive treatment. In the elderly group, 4 patients required long-term antibiotic suppressive treatment and one further patient required second GS (Table 3). Other general complications included the necessity of transfusion in 14 cases (31.1%). Furthermore, one periprosthetic fracture (femoral shaft fracture Vancouver C) was identified after stage-two surgery (2.3%). One complication in each the mid-aged and elderly population group was observed (Table 3).

**Table 3:** Age-group-specific success rate of treatment using Girdlestone

	Group 1 (32–44)	Group 2 (45–57)	Group 3 (58–70)
*n*, %	4 (%)	9 (%)	31 (%)
Successful treatment	4 (100)	7 (77.8)	26 (83.9)
Treatment failure	0 (0)	2 (22.2)	5 (16.1)
Transfusions	0 (0)	4 (44.4)	10 (32.3)
Periprosthetic fracture	0 (0)	0 (0)	1 (3.2)

No other complications were found such as muscle contracture or fracture during GS. In all cases, a THA revision implantation was performed after the successful eradication using girdlestone arthroplasty.

### Successful treatment using a spacer

**Fig. 1:**
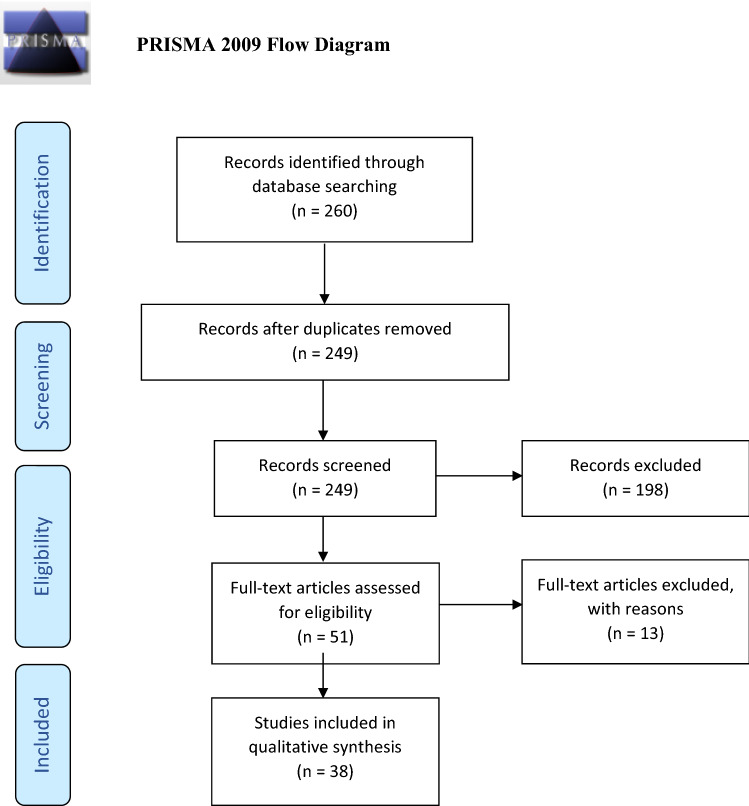
Included articles according to the PRISMA guidelines

For the eradication rate following a two-stage procedure with a spacer, we performed a systematic review (Fig. 1). A total of 38 articles were included [9, 21–57] analyzing 9, 428 patients undergoing two-stage revision using molded with or without endoskeleton femoral or articulating antibiotic loaded spacers. The mean age was 66.0 ± 6.6 years and therefore comparable to our data with 59.5 years. The overall success in treatment was 86.5 ± 19.5% (range 61–100%). In 2.4% of cases, a girdlestone procedure was required after spacer implantation, and in 12.5%, a reimplantation of a new THA was not possible. No differentiation among age was performed. Additionally, the complication rate for spacers was 20.4 ± 16.4%. More precisely, the dislocation rate was 6.8 ± 4.7%, spacer fractures were seen in 3.7 ± 4.5%, the spacer loosening rate was 1.5 ± 2.8%, and the rate for periprosthetic femoral factures was 6.9 ± 9.5%. All studies included are illustrated in Table 4 (Supplemental Material).

## Discussion

With regards of the increasing necessity of revision surgeries for chronic PJI, the successful treatment strategies are at highest importance. Even though two-stage exchange strategies are practiced for decades, no gold standard of treatment has been established with cure rates of treatment being reported in a wide range (76–100%) [16, 58, 59]. Up to now, a variety of different salvage techniques have been described with Sp being the most frequently implanted device in a two-stage procedure [4, 7].

This study shows that treatment cure rate, as defined above, using GS is close to equal to the usage of Sp and in total, represents less material-related complications.

When comparing our data with the data using Sp as in our systematic review, the patients’ age was approximately equivalent with 66.0 years for Sp compared to 59.3 years for GS. Likewise, the cure rate was close to equal with 84.1% for GS, respectively, 86.5 ± 19.5% for Sp. In a study, a high infection eradiation rate was achieved for GS if a multidisciplinary, patient individual treatment was established which should acquire an individual algorithm for each patient. Additionally a short antibiofilm-active agent period prior reimplantation was suggested [16]. In our study, this concept was followed resulting in all patients undergoing reimplantation at a mean of 69 ± 34.2 days after stage-one surgery.

Implantation of temporary Sp between stage-one and stage-two surgery is commonly used to prevent the disadvantages of a GS procedure, meaning that Sp enable preservation of the joint space and reduces dead space, to prevent soft-tissue contractures which can lead to leg length shortage and in cases of an antibiotic loaded spacer, ensure high local concentration of antibiotic.

However, the main disadvantages for the usage of Sp are the high risk of spacer-related complications ranging from 0 to 81.6% based on the systematic review of the literature. Especially, the spacer dislocation described with up to 17.0% of cases, periprosthetic fractures (up to 40%), and spacer fractures (up to 13.7%) need to be considered [7, 45, 53, 60]. Additionally, one further downside when using Sp is the reimplantation that cannot always be achieved and therefore resulting in a spacer-retaining rate of 12.5 ± 18.0%. However, with newer surgical techniques using custom-made articulating spacers, complication rates might be lower and patients frequently achieve a low pain level and good mobility already during the spacer period. [54]

In our GS group, we show that the complication rate is 2.3% including one periprosthetic fracture. Furthermore, all patients were able to undergo stage-two surgery with implantation of a new prosthesis. When looking at the prosthesis-free interval, the GS group resulted in reimplantation at around 6–10 weeks compared to 5 months according to the data of the review of the literature from Rava et al*. *[7].

There are several limitations to this study. First, it is of retrospective design of a prospectively enrolled cohort with a follow-up of 24 months. This does not allow to conclude on long-term complications. Furthermore, our study has a relatively small sample size at a single center, and there might have been unmeasured factors which led to selection bias and therefore limits the generalizability of our results. Moreover, for Sp implants, we only had data from the literature due to the fact that our center rarely performs implantation of Sp. Furthermore, our data did not include pre- or postoperative outcome parameters.

## Conclusion

Girdlestone procedure shows an acceptable success rate in treatment of chronic PJI compared to Sp with a low complication rate. Moreover, in situations where an Sp cannot be rooted with a sufficient degree of security or where the risk of a dislocation is high, GS is an alternative to Spacer treatment.

## Supplementary Information

Below is the link to the electronic supplementary material.

Supplementary file1 (DOCX 26 KB)
