# Supplementary material for: Cure rate of infections is not an argument for spacer in two-stage revision arthroplasty of the hip
Source: Arch Orthop Trauma Surg. 2022 May 9;143(4):2199–207. doi: 10.1007/s00402-022-04463-9 (PMC10030410; doi:10.1007/s00402-022-04463-9)
Supplement: Supplementary file 1 — Supplementary file1 (DOCX 26 KB) [file 402_2022_4463_MOESM1_ESM.docx]

Table 4: Systematic review and included articles – spacer retaining rate where no stage two procedure was performed.

| **Authors** | **n (hips)** | **Mean age (years)** | **Mean follow-up (months)** | **Infection eradication rate (%)** | **Complication rate (%)** | **Deaths (%)** | **Dislocation rate (%)** | **Spacer** | **Girdle stone required %** | **Spacer retaining rate %** | **Spacer fracture rate** | **Loosening rate %** | **Periprosthetic femoral fractures rate %** |
| --- | --- | --- | --- | --- | --- | --- | --- | --- | --- | --- | --- | --- | --- |
| Anagnostakos K et al 2016 | 22 | 59.7 | 44.8 | 87 | 23 |  | 9 | Molded+ endoskeleton |  | 27.3 | 13.7 | 0 | 0 |
| Beaupre LA et al 2017 | 29 | 70.8 | 24 | 86 | - |  | - | Prostalac | 6.8 | 68 |  |  |  |
| Ben-Lulu O et al 2012 | 11 | 79 | 12 | 91 | 9 |  | 9 | Molded+ endoskeleton+intramedullary nail | - |  | 0 | 9 | 0 |
| Bialecki J et al 2020 | 71 | 59.3 | 5.8 | 93.1 | 14.1 | 0 | 1.4 | Molded+ endoskeleton |  |  |  |  | 5.6 |
| Bori G et al 2014 | 74 | 70 | 53.18 | 61 | 10.81 |  | 10.81 | Spacer G | 0 | 13.51 |  |  |  |
| Burastero G et al 2017 (Full spacer) | 17 | 68 | 33.2 | 96.8 | - |  | 3.2 | Full molded+ endoskeleton spacer |  |  | 3.2 |  | 3.2 |
| Burastero G et al 2017 Femoral spacer | 40 | 67 | 45.3 | 92.5 |  |  | 10 | Femoral molded+ endoskeleton spacer |  |  | 0 |  | 0 |
| Cabrita HB et al 2007 | 38 | 54.6 | 48 | 89.1 | 81.6 | 2.6 | 7.9 | Vancomycin loaded spacer group |  |  | 2.6 | 2.6 | 13.2 |
| Cancienne JM et al 2017 | 7146 | - | 12 | 93.5 |  | 6.5 | - | - | 5.7 | 16.8 |  |  |  |
| Chalmers BP et al 2018 | 135 | 65 | 42 | 92 | 7 | 5.2 | 5 | Molded+ endoskeleton |  |  |  |  | 2 |
| Faschingbauer M et al 2015 | 138 | 69.3 |  | - | 19.6 |  | 8.7 | Molded+ endoskeleton |  |  | 8.7 |  | 9.4 |
| Fleck EE et al 2011 | 14 | 60.8 | 50 | 100 | 0 |  | - | Molded+ endoskeleton | 0 | 0 | 0 | 0 | 0 |
| Fu J et al 2020 | 26 | 46.7 | 49.2 | 96.2 | 19.2 |  | 3.8 | Molded+ endoskeleton |  | 3.9 |  | 3.9 | 3.9 |
| Gomez MM et al 2015 | 178 | 64.6 | 56.2 | 81.7 | - |  | - | Molded+ endoskeleton | 4.6 | 40.4 |  |  |  |
| Grammatopoulos G et al 2017 | 66 | 67 | 96 | 89 | 29 |  | 17 | - |  | 0 | 0 | 0 | 0 |
| Hsieh PH et al 2004 | 58 |  | 58.8 | 96.6 | 12.1 |  | 3.4 | - |  |  | 3.4 |  |  |
| Jones CW et al 2019 | 185 | 64 |  | 96 | 26 | 4 | 9 | Molded |  |  | 8 |  | 7 |
| Jung et al 2009 | 82 | 70 | 54 | 91 | 49.8 |  | 17 | Custom-made |  |  | 10.2 |  | 13.6 |
| Kelm et al 2009 | 10 | 66 | 12 | 100 | 10 |  | 10 | Molded | - | 20 | 0 | 0 | 0 |
| Kipp JO et al 2019 | 29 | 72.5 | 6 | 93.1 | 27.6 | 13.8 | 3.5 | Molded+ endoskeleton | 6.9 | 0 | 6.9 |  |  |
| Lausmann C et al 2018 | 30 | 69.8 |  | 96.6 | 16.7 |  | 6.7 | Molded |  |  |  |  | 3.4 |
| Lee WY et al 2017 | 20 | 67.7 | 68.8 | 94.9 | - |  | - | Prostalac | 0 | 0 |  |  |  |
| Lombardi AV Jr et al 2014 | 7 | 66.8 | 19.2 | 86 | 0 | 14 | 0 | Molded+ endoskeleton | 0 | 0 | 0 | 0 | 0 |
| Lunz A et al 2021 | 24 | 70 | 19 | 95.8 | 12.5 |  | 4.2 | Articulating Spacer | 0 | 0 | 0 | 0 | 0 |
| Marczak D et al 2017 | 47 | 62 | 24 | 91 | 12.7 |  | 2.12 | Vancogenx Spacer | 0 | 0 | 0 | 0 | 0 |
| Masri BA et al 2007 | 31 | 65 | 47 | 90 | - |  | - | Prostalac |  |  |  |  |  |
| Matar HE et al 2019 | 29 | 63 | 60 | 96.5 | 13.8 |  | 3.4 | Antibiotic-loaded molded |  |  |  |  |  |
| Mederake M et al 2021 | 130 | 71 | 51 | 92 | 10 | 5.4 | 8 | Molded | - | - | 1 |  | 1 |
| Nahhas CR et al 2021 | 20 | 58.2 | 36 | 85 | 15 | 0 | 5 | Mobile Molded+ endoskeleton |  | 7.7 |  |  |  |
| Petis SM et al 2019 | 164 | 68.3 | 145.2 | 85.1 | 35.4 |  | 12 | Molded |  |  |  | 0.6 |  |
| Quayle J et al 2021 | 21 | 72.4 | 46.8 | 71.4 | 33.3 | 9.5 | 9.5 | Long femoral spacers |  |  | 9.5 |  | 14 |
| Rollo G et al 2020 (Handmade) | 24 | 79.7 | 26.8 | 100 | 29.2 |  | 0 | Handmade Spacer | 0 | 0 |  |  | 25 |
| Rollo G et al 2020 (Molded) | 26 | 76.8 | 26.3 | 100 | 30.8 |  | 7.7 | Molded+ endoskeleton |  |  |  |  | 7.7 |
| Romanò CL et al 2011 | 20 | 55.7 | 56.6 | 95 | 0 |  | 0 | Interspace | 0 | 0 |  |  |  |
| Romanò CL et al 2012 | 183 | 60.3 | 24 | 96.4 | 20.4 |  | 16.4 | Interspace | 3.3 | 0 | 0 | 0 | 0 |
| Staats K et al 2017 | 25 | 57 | 24 | 100 | 60 |  | - | Molded+ endoskeleton |  |  |  |  |  |
| Tsung JD et al 2014 | 76 | 71.5 | 80.4 | 84.2 | 22.4 | 1.3 | 10.5 | Articulating Spacer |  |  |  |  | 7.9 |
| Uchiyama K et al 2013 | 36 | 62.4 | 48.6 | 91.6 |  |  | 2.8 | Molded+ endoskeleton |  | 13.9 |  |  |  |
| Veltman ES et al 2020 (Handmolded articulating spacer) | 15 | 66 | 24 | 93 | - |  | 6.7 | Articulating spacer | 6.7 |  |  |  | 40 |
| Veltman ES et al 2020 (Prefabricated spacer) | 55 | 68 | 51 | 78 | - | 18.2 | 1.8 | Prefabricated spacer |  |  |  |  | 16.4 |
| Vielgut I et al 2015 | 76 | 66.5 | 20.5 | 70 | - |  | - | Molded+ endoskeleton | 0 | 26.6 |  |  |  |
|  |  |  |  |  |  |  |  |  |  |  |  |  |  |
| **Total** | **9428** | **66.0** | **42.1** | **86.5** | **20.4** | **6.7** | **6.8** |  | **2.4** | **12.5** | **3.7** | **1.5** | **6.9** |
| **SD** |  | **6.6** | **26.8** | **19.5** | **16.4** | **6.0** | **4.7** |  | **3.0** | **18.0** | **4.5** | **2.8** | **9.5.3** |
